# A Randomized-Controlled Trial Examining the Effects of Reflexology on Anxiety of Patients Undergoing Coronary Angiography

**DOI:** 10.5812/nms.12167

**Published:** 2013-09-15

**Authors:** Mehdi Molavi Vardanjani, Negin Masoudi Alavi, Narges Sadat Razavi, Mohammad Aghajani, Esmail Azizi-Fini, Seied Morteza Vaghefi

**Affiliations:** 1 Student Search Committee, Kashan University of Medical Sciences, Kashan, IR Iran; 2 Trauma Nursing Research Center, Kashan University of Medical Sciences, Kashan, IR Iran; 3 Department of Cardiology, Kashan University of Medical Sciences, Kashan, IR Iran; 4 Shahid Beheshti Hospital, Kashan University of Medical Sciences, Kashan, IR Iran

**Keywords:** Foot massage, Anxiety, Coronary angiography

## Abstract

**Background:**

The anxiety reduction before coronary angiography has clinical advantages and is one of the objectives of nursing. Reflexology is a non-invasive method that has been used in several clinical situations. Applying reflexology might have effect on the reduction of anxiety before coronary angiography.

**Objectives:**

The aim of this randomized clinical trial was to investigate the effect of reflexology on anxiety among patients undergoing coronary angiography.

**Patients and Methods:**

This trial was conducted in Shahid Beheshti Hospital, in Kashan, Iran. One hundred male patients who were undergoing coronary angiography were randomly enrolled into intervention and placebo groups. The intervention protocol was included 30 minutes of general foot massage and the stimulation of three reflex points including solar plexus, pituitary gland, and heart. The placebo group only received the general foot massage. Spielbergers state trait anxiety inventory was used to assess the anxiety experienced by patients. Data was analyzed using Man-Witney, Wilcoxon and Chi-square tests. The stepwise multiple regressions used to analyze the variables that are involved in anxiety reduction.

**Results:**

The mean range of anxiety decreased from 53.24 to 45.24 in reflexology group which represented 8 score reduction (P = 0.0001). The reduction in anxiety was 5.9 score in placebo group which was also significant (P = 0.0001). The anxiety reduction was significantly higher in reflexology group (P = 0.014). The stepwise multiple regression analysis showed that doing reflexology can explain the 7.5% of anxiety reduction which made a significant model.

**Conclusions:**

Reflexology can decrease the anxiety level before coronary angiography. Therefore, reflexology before coronary angiography is recommended.

## 1. Background

Coronary artery disease is one of the most common reasons of death all over the world and coronary angiography (CA) is used broadly for evaluation and treatment of this disorder (1). It was estimated that a total of 260514 coronary angiography (347 per 100000 persons), was performed in Iran in 2011. CA is the fourth most common invasive treatments in Iran (2).

Coronary angiography has been shown to cause anxiety, fear and emotional stress. More than 80% of patients reported anxiety before CA (3). Patients being scheduled for CA have demonstrated an increased anxiety during the waiting period (4). Fifty percent of patients have reported that their fears, anxiety and uncertainty were more distressing than their chest pain (3). Interestingly, the anxiety before CA was reported more, than the anxiety before open heart surgery. The reason might be that the patients were less prepared for this procedure compared with open heart surgery. Furthermore, the increased anxiety prior to surgical intervention, leads to higher complication rates during and after the surgical procedures (3).

Different approaches have been used for reducing the stress and anxiety level prior to CA. Educational interventions, music therapy, massage, guided imagery, therapeutic touches and stress management techniques revealed positive effects on anxiety before CA (5). One study demonstrated that a 20-minute back massage before CA was significantly reduced the systolic blood pressure (6). Although a 10-minute massage before CA procedure, was not sufficient to decrease stress level (7). No significant reduction of anxiety was found in Chinese patients undergoing CA following sensory information and music therapy (8). Astley et al. showed that applying audiovisual educational techniques had no effect on anxiety prior to CA (9). A patient-controlled study, revealed that music therapy had no significant effect on anxiety level before and after CA (10).

Complementary and alternative medicine including reflexology interventions have widespread acceptance, largely without the clinical evidence for safety and efficacy (11). Reﬂexology is a popular complementary and alternative medicine, which is based upon the application of pressure to specific reﬂex points of the hands and feet, corresponding with specific areas of the body (12). It has been reported that the specific massage to these points, increases the blood supply to the corresponding organs (13). Although the exact mechanism of action underlying the reﬂexology is unknown, several theories exist. Reﬂexology is suggested to relieve tension and increase the blood supply (12). It is stated that Reﬂexology activates the body’s own healing power (14).

Nakamaru et al. examined the relationship between cortical activity and sensory stimulation of reﬂexed areas using functional magnetic resonance imaging. They found that stimulation of three reﬂexed areas on the left foot, relating to the eye, shoulder, and small intestine activated not only the somatosensory areas corresponding to the foot, but also activate the corresponding somatosensory areas to the eye, shoulder, and small intestine or neighboring body parts (15). A research in healthy volunteers showed that reﬂexology did not affect the heart rate, systolic and diastolic blood pressure, however analysis indicated that reﬂexology appeared to affect cardiac index to a significant level (13).

While evidences that support the clinical effectiveness of reﬂexology are limited, a small number of studies have reported the benefits of this technique on relieving the low back pain (12), fibromyalgia symptoms (14), increasing peak expiratory flow in chronic obstructive pulmonary disease (16), reducing pain and duration of labor (17), and improving the physical effectiveness in patients with advanced breast cancer (11). In a systematic review, Wang et al. found no evidence for any effect of reﬂexology in any conditions, with the exception of urinary symptoms associated with multiple sclerosis (18). In a cross-over experimental study with healthy volunteers, reﬂexology had a powerful anxiety-reduction effect although baseline salivary cortisol and melatonin were not significantly changed after reﬂexology (19).

The reduction of anxiety before CA has clinical advantages and is one of the objectives of nursing. Some methods have been recommended for relieving anxiety before CA but none of them are evidence-based. Reflexology is a non-invasive method that has been used in several clinical situations. Applying reflexology might have effect on the reduction of anxiety level before CA.

## 2. Objective

Since the randomized clinical trial (RCT) is the gold standard for research by which health professionals make clinical decisions (11). The aim of this RCT was to investigate the effect of reflexology on anxiety of patients undergoing coronary angiography.

## 3. Patients and Methods

**Figure 1.**
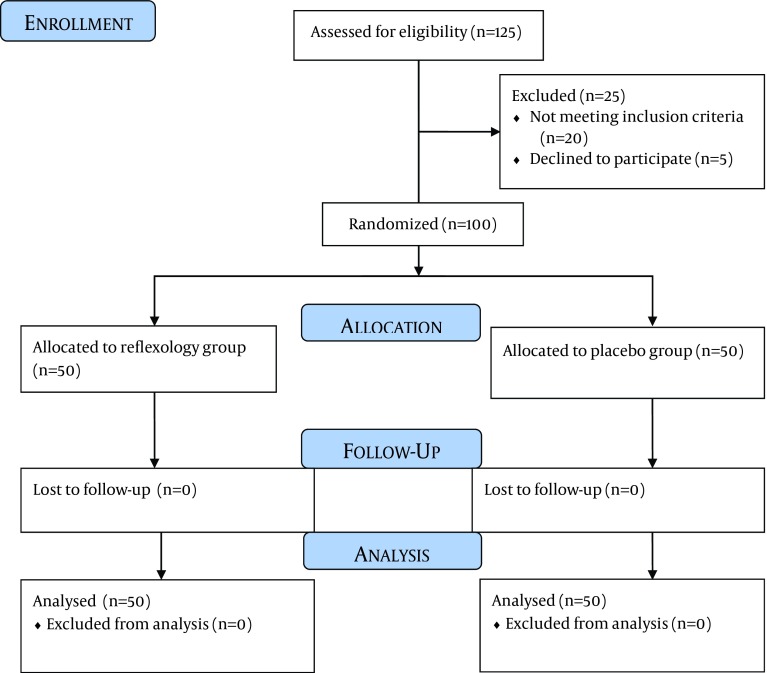
The Sampling Framework of the Study

This single blind RCT was conducted in Shahid Beheshti Hospital, Kashan, Iran. The sample size was consisted of 50 patients in each group, using the formula for interventional studies. The study assumptions were: the difference between the means anxiety in groups is six and the standard deviation is 10, the confidence of 95% and power of 80% ( 20 ). One hundred male patients who were undergoing coronary angiography between September 2012 and February 2013 were enrolled into the study. All the patients were fully conscious, and had no history of depressive or anxiety disorders or consumption of anti-anxiety drugs 48 hours before the study. The patients were candidate for their first elective coronary angiography. Emergency angiography cases and patients with the symptoms of myocardial infarction were excluded from this study. The patients were reflexology-naive and had normal lower limbs (no previous operation including varicose vein, peripheral neuropathy or deep vein thrombosis). Only male patients were recruited in order to eliminate the gender influence on anxiety. The patients were randomized by coin tossing to reflexology or placebo group. The framework of sampling is shown in Figure 1. 

All the interventions in both groups were performed by one person (the first author) in the morning shift. The procedure was explained to the subjects and the written informed consents obtained. The intervention protocol was established including, general foot massage for relaxation in both groups and stimulation of three reflexology areas of reflexology group. One day before angiography the reflexology group received a reflexology treatment for 30 minutes. Patients were treated in a near supine position in a quiet room of angiography ward, Shahid Beheshti hospital. At the beginning patients received the general foot massage at both feet in the following order:

- The therapist washed his hands with warm water and moderate amount of sunflower oil was applied to the hands.
- The foot was massaged from ankle to toe with moderate pressure using both hands for 10 times.
- The foot was hold with one hand and the other hand rotated the foot at the ankle. This procedure repeated eight times.

**Figure 2.**
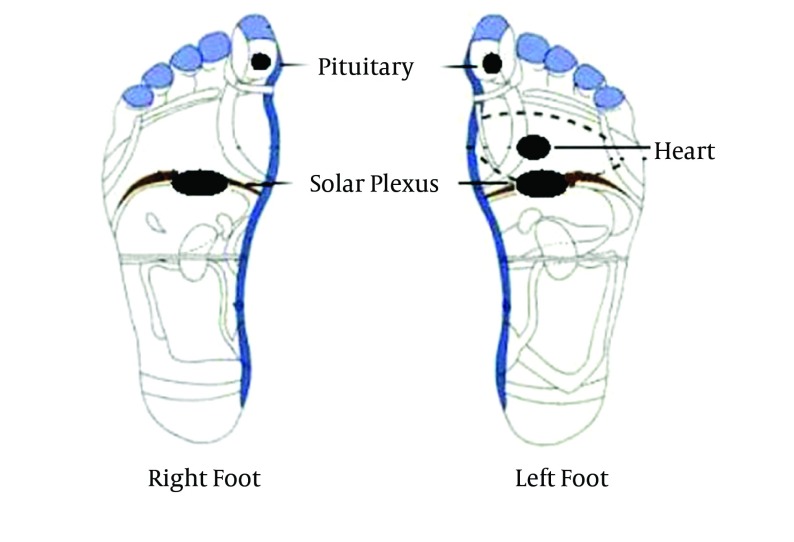
The Location of Reflexology Points Used in this Research

After general foot massage, the three reflexology areas of solar plexus, pituitary gland, and heart were used for stimulation (Figure 2). The solar plexus and pituitary points were located according to the provided plot by Reflexologists, and the heart point located according to Jones et al. recommendation ( 21 ). The reflexologist exerted firm downward pressure with his thumb in the areas for two minutes in every area. The pressure regulated in a way that made the upper thumb white but patients did not feel any pain. Then a circle massage was applied to the specific points. The placebo group received the general foot massage without stimulation of the reflexology points. 

The Spielbergers state-trait anxiety inventory (STAI) was used to assess changes in transitory anxiety experienced by patients prior to and following treatment. The STAI is a well-validated tool that can differentiate the temporary condition of ‘‘state anxiety’’ and the more general quality of ‘‘trait anxiety. The STAI has a likert scale on four levels of anxiety intensity from ‘not at all’ to ‘very much’. The sum of 20 items score between 20 and 80 (22). STAI has been translated and used in Iran with areliability of 0.86 (23).These scores increase in response to psychological stresses. The anxiety levels were recorded at the beginning and 30 minutes after intervention. The person who recorded the STAI was not aware of the kind of interventional group.

### 3.1. Ethical Consideration

The Helsinki declaration was respected in this study. All participants were informed of the details of the study and gave their written informed consent. The participants were free to leave the study at any time and the personal information was kept secure. The study was approved by the Ethical Committee of Kashan University of Medical Sciences.

### 3.2. Statistical Analysis

All data were analyzed using Statistical Package for the Social Sciences (SPSS; Version 16). The normality of the data was analyzed using Kolmogorov-Smirnov test. The data for anxiety and other quantitative variables such as age were compared using Man-Witney U test. Pre and post-treatment change within groups was analyzed using Wilcoxon test. The chi-square test was used for comparing the qualitative variables within the groups, such as education, and the history of myocardial infarction. The stepwise multiple regressions used to analyze the variables that explain the anxiety reduction.

## 4. Results

The average age was 52.6 ± 7.5 in intervention and 54.8 ± 5.6 in placebo group. The intervention and placebo group did not show significant differences in their demographic features (Table 1). 

The mean anxiety level was significantly higher in reflexology group before the intervention, although it was not significantly different in the groups after interventions (Table 2). 

**Table 1.** Demographic Composition of the Two Randomly Assigned Groups

	Groups		P value
	Reflexology	Placebo	
**Age, y, Mean ± SD**	52.6 ± 7.8	54.8 ± 5.6	0.203
**The duration of cardiac disease, y, Mean ± SD**	2.8 ± 3.7	3.6 ± 3.5	0.099
**The history of myocardial infarction, No. (%)**			0.137
Yes	4 (8)	9 (18)	
No	46 (92)	41 (82)	
**Education, No. (%)**			0.057
Illiterate	18 (36)	13 (26)	
Primary	12 (24)	26 (52)	
Secondary	5 (10)	3 (6)	
Diploma	11 (22)	7 (14)	
Academic	4 (8)	1 (2)	
**Marital status, No. (%)**			0.72
Married	45 (90)	47 (94)	
Single or widowed	5 (10)	3 (6)	
**History of surgery**			0.057
Yes	40 (80)	32 (64)	
No	10 (20)	18 (36)	
**Occupational status, No. (%)**			0.656
Occupied	35 (70)	37 (74)	
Not occupied	15 (30)	13 (26)	

**Table 2.** The Anxiety Level in Reflexology and Placebo Groups

**Anxiety**	**Groups**		**Z**	**Pvalue**
	**Reflexology**	**Placebo**		
**Before intervention**	53.24 ± 4.29	49.62 ± 5.31	-3.733	0.000
**After intervention**	45.24 ± 3.32	43.7 ± 5.06	-.850	0.395
**Amount of anxiety reduction**	8 ± 3.8	5.9 ± 3.5	-2.468	0.014

The mean amount of anxiety decreased from 53.24 to 45.24 in reflexology group which represented an eight-score reduction. The Wilcoxon Signed Ranked Test showed significant reduction in anxiety among reflexology group patients (Z = -6.1, P = 0.0001). The reduction in anxiety was 5.9 in placebo group which was also significant (Z = -5.738, P = 0.0001). The Mann-Witney U test showed that the anxiety reduction was significantly higher in reflexology group (Z = -2.468, P =0.014). 

The stepwise multiple regression analysis showed that reflextion can explain the 7.5% of anxiety reduction which made a significant model (R ^2 ^=0.075, Adjusted R ^2 ^= 0.066, β = -0.274, P = 0.006). Other variables such as age, history of MI, education, duration of the problem and the occupational status did not show any significant relation with the anxiety reduction (Table 3). 

**Table 3.** The Multiple Regression of Dependent Variable of Anxiety Reduction and Other Excluded Variables

**Variables**	ß	**t**	P	**95% Confidence Interval for B**	
				**Upper Bound**	**Lower Bound**
**Age**	-0.084	-0.847	0.399	-0.285	0.175
**Duration of problem**	0.019	0.192	0.848	-0.120	0.426
**History of MI**	0.091	0.920	0.360	-1.241	4.787
**Job**	-0.083	-0.843	0.401	-2.261	2.227
**Education**	0.056	0.572	0.569	-1.161	1.138

## 5. Discussion

The objective of the present study was to determine the effect of reflexology on anxiety level before CA. The results showed that both general foot massage and reflexology could decrease the anxiety level before CA. The anxiety reduction was significantly higher in reflexology group. This reduction was considerably higher than the placebo group who simply received general foot massage.

Some experts believe that local finger pressure on reflex points of the feet can affect the function of target organs and promote relaxation and healing responses, therefore a range of symptoms can be treated by reﬂexology (13, 19). McVicar et al. in a study on the effect of reflexolgy on anxiety, showed that reflexology intervention decreased the state anxiety but no evidence for its influence on the ‘trait’ anxiety was reported (19), that was an predictable result, since trait anxiety, unlike state type, is not a transitory state and needs a long time intervention. In this research also the anxiety reduced after reflexology. From a methodological viewpoint, the use of questionnaires that can record the state anxiety in researches using reflexology as the intervention is recommended.

Another study designed to evaluate the effect of reflexology on mental stress induced tests in healthy individuals. The results showed that there were significant reductions in systolic and diastolic blood pressure following reflexology (24). Lu et al. investigated the effect of foot reflexing on the autonomic nervous modulation in patients with coronary artery disease. He found that reflexology can increase the vagal modulation and decrease the blood pressure in patients with coronary artery disease (25).

However, Wang et al. analyzed five Randomized Clinical Trials, and found that reﬂexology does not have extra beneficial effect compared to the non-specific effects produced by foot massage (18). Also in a systematic review conducted by Edzard, convincing evidences suggesting that reflexology has health benefits over a placebo response were not observed, he concluded that there were limited RCTs in this field and the methodological quality of the primary studies was often poor. Most high-quality RCTs were not effective (26).

The anxiety mean range was 51.43 ± 5.1 before intervention which is much less compared to the 91.42 ± 21.2 reported in a study conducted by Jamali et al. on patients with irritable bowel Syndrome in Shahid Beheshti Hospital in 2010 (27). It might show that the overall anxiety in these patients might be less than expected amount, although this conclusion needs further comparative studies.

When we are studying about the complementary medicine including reflexology, we should consider that some experts believe there are some adverse effects in the beginning of interventions, which are often known as "healing crisis". Healing crises frequently happened during and immediately after the treatment with the following manifestations: localized or distal pain, perspiration or shivering and changes in the heart rate. This phenomenon is also described as "cleansing process", since it is believed that the treatment activates the body healing power, where accumulated waste products and toxins released into the bloodstream. These adverse reactions resolve after seventh and eighth sessions. So these interventions should be provided in more than one session; consequently, the benefits can be assessed and documented (14, 28). In current study we just performed reflexology in one session which might be a limitation of our study.

Another methodological challenge in this study is a surprising lack of consistency of reﬂex point location in published reﬂexology foot maps. Many different types of reﬂexology maps are available, produced by a range of map or chart providers. Any reﬂexologist training provider can create and publish a map according to their own intuitive interpretation of where the various reﬂex points are located. Especially the heart reﬂex point has been placed in different areas (13, 21). An other limitation of this study is that it cannot be determined whether the effects are resulted from the reflexology alone or the impact of reﬂexology and massage are combined. Only Applying massage can have hemodynamic effects (29). In reflexology interventions it is difficult to provide controls for adequate comparative purposes. Many studies used placebo reﬂexology treatment in the form of general foot massage as study control (13). We also used the same control method. This is difficult to see how simple foot massage, as a control method, can be differentiated from reﬂexology foot massage, particularly in terms of avoiding working any of the planter reﬂex points during the manual manipulation which may be a limitation of this study.

It showed that two groups of this trial may not have the same condition at the start (as shown by the differences in the anxiety level before CA), despite random allocation into two groups, and the similarity of the demographic variables.

With the increasing usage of complementary and alternative medicine (CAM) such as massage therapy, acupressure and reflexology by the general public, it is important that healthcare professionals, including nurses, make informed decisions when advising clients who referred to CAM providers (30, 31). Nurses educated in providing holistic healing modalities including reflexology should be considered at every health-care setting. A holistic nurse is defined as a nurse who recognizes and integrates body–mind–spirit principles and modalities in daily life and clinical practice. Good clinical practice in reﬂexology needs to incorporate recommendations from best clinical practice experiences and research findings.

In conclusion this study shows that reflexology can decrease the anxiety level before CA. Therefore, the use of reflexology in coronary angiography units is recommended. Reflexology is a relatively new area of care in cardiac patients. It is a simple and convenient technique that requires no tools, although its application needs special training. This was a small-scale study with some limitations so further researches with larger sample size is needed in this area. The effect of reflexology on the complications of CA also can be studied. The effects of reflexology in two and more sessions can be compared in further studies.
